# A safe seal: controlled flow arrest with dual balloon protection for embolisation of a large spontaneous splenic arteriovenous fistula—a case report

**DOI:** 10.1186/s42155-026-00669-9

**Published:** 2026-04-16

**Authors:** Preethi Vijayasekar, Tejesh Jagannathan, Karthik Kulanthaivelu, Sabarish Sekar, Ansan Joseph

**Affiliations:** Department of Neuro and Interventional Radiology, Sri Ramachandra Institute of Higher Education and Research, Chennai, 600116 India

**Keywords:** Splenic artery aneurysm, Splenic arteriovenous fistula, Dual balloon assisted embolisation

## Abstract

**Background:**

Splenic artery aneurysm (SAA) with splenic arteriovenous fistula (SAVF) is rare. Endovascular embolisation offers a minimally invasive, spleen-preserving alternative to surgery, particularly in patients with connective tissue disorders. This case report describes a technically challenging proximal SAA with high-flow SAVF successfully treated using dual-balloon-assisted coil and N-butyl cyanoacrylate (NBCA) glue embolisation, with special consideration for vascular Ehlers–Danlos syndrome (vEDS).

**Case presentation:**

A 28-year-old short-statured woman with micrognathia had computed tomography (CT) showing a partially thrombosed proximal SAA and early arterial-phase enhancement of portomesenteric veins. Given her young age and constitutional features, an underlying connective-tissue vasculopathy such as vEDS was suspected. Dual-balloon flow control was achieved using a 6 × 15 mm Eclipse balloon at the hepatic–coeliac bifurcation (arterial inflow control) and a 12 × 40 mm Mustang balloon in the splenic vein (venous outflow control), followed by dense coil packing and controlled NBCA injection. Post-embolisation angiography and follow-up CT demonstrated exclusion of the aneurysm–fistula complex with preserved hepatic and portal venous flows and viable splenic parenchyma.

**Conclusion:**

Dual-balloon-assisted coil and N-butyl cyanoacrylate (NBCA) embolisation enables precise, controlled flow arrest in high-flow SAA–SAVF, minimising non-target embolisation. This case highlights its value as a safe, spleen-preserving, and durable option—particularly relevant in patients with suspected connective-tissue disorders such as vEDS, where arterial fragility mandates meticulous endovascular technique.

**Level of evidence:**

4 (Case Report).

**Graphical Abstract:**

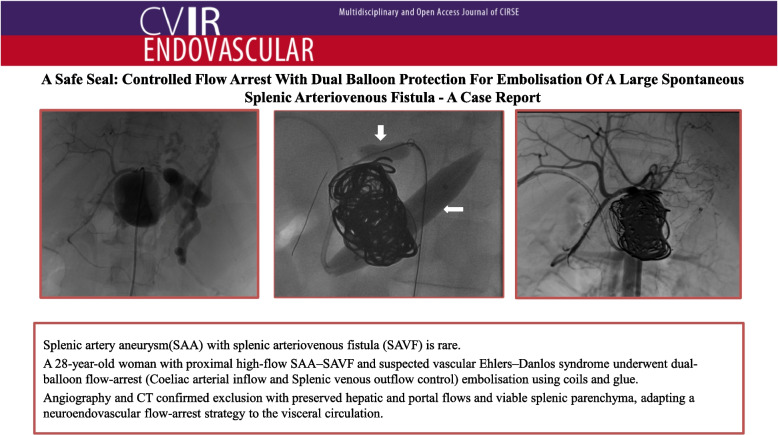

## Introduction

Splenic artery aneurysm (SAA) represents approximately 60% of all visceral artery aneurysms, whereas splenic arteriovenous fistula (SAVF) is exceedingly rare [1]. Coexistence of these lesions—often due to aneurysmal rupture into the splenic vein—can result in portal hypertension, splenomegaly, and high-output cardiac failure. Spontaneous SAVFs occur predominantly in women [2], and are associated with SAAs in more than half of cases [2]. SAAs most commonly arise from the distal third of the splenic artery (74–87%). Although splenectomy was historically the standard therapy, endovascular (EV) management now allows spleen preservation with low morbidity. This report describes a technically challenging proximal SAA with SAVF successfully treated by dual-balloon-assisted coil and glue embolisation.

## Case report

A 28-year-old short-statured, thin-built woman presented with left upper-quadrant abdominal pain for two months, worsening over the preceding week. There were no specific aggravating or relieving factors. Clinical examination revealed micrognathia without joint laxity or skin hyperextensibility.

Ultrasound of the abdomen showed splenomegaly (12.7 × 5 cm), with markedly hypoechoic parenchyma and absent intraparenchymal vascular flow on colour Doppler, suggesting splenic infarction or devascularization. A well-defined aneurysm measuring approximately 4 × 3.5 cm with internal turbulent colour flow was noted in the epigastric region, likely arising from the splenic artery.

**Fig. 1:**
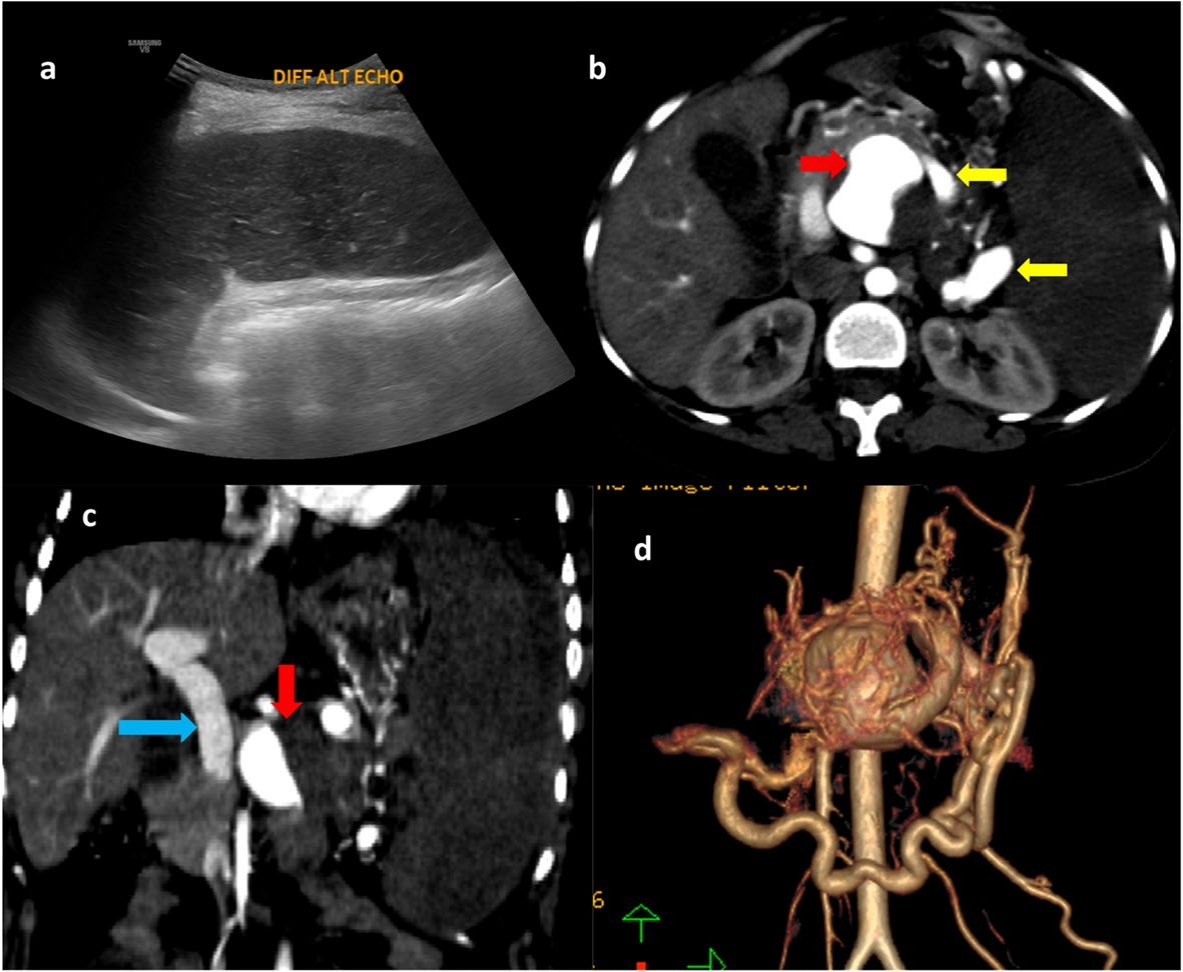
**A** Ultrasound of the abdomen shows a markedly hypoechoic spleen. **B**, **C** Axial and coronal CECT images demonstrate a partially thrombosed splenic artery aneurysm (red arrow) with early arterial opacification of the splenic vein (yellow arrows in **B**) and portal vein (blue arrow in **C**), consistent with a splenic arteriovenous fistula. **D** 3D volume-rendered reconstruction depicts the splenic artery aneurysm with markedly dilated splenic (arrow) and gastric veins

Multi-phase contrast-enhanced CT (CECT) of the abdomen demonstrated a partially thrombosed aneurysm near the origin of the splenic artery, with early arterial-phase enhancement of the portomesenteric veins, suggestive of a high-flow splenic arteriovenous fistula (SAVF) (Fig. 1). The spleen was enlarged, showing subtle parenchymal enhancement limited to the upper pole and preserved capsular enhancement, indicating compromised splenic perfusion secondary to shunting.

Given the patient’s constitutional abnormalities and the presence of a visceral arterial aneurysm at a young age, a syndromic connective-tissue disorder (e.g. Ehlers–Danlos variant or related vasculopathy) was considered rather. With these considerations, the patient was carefully planned and prepared for endovascular management using meticulous technique and minimal instrumentation to reduce procedural risk.

Under monitored anaesthesia care, right common femoral arterial access was achieved via the retrograde Seldinger technique. A 5-F Cobra catheter advanced into the coeliac artery demonstrated a proximal large SAA measuring 4.1 × 3.9 × 3.6 cm with early splenic and portal venous opacification and non-opacification of the distal splenic artery. An 8F guide catheter was positioned in the coeliac trunk. Through a percutaneous puncture of the right segment V portal branch, a 7-F sheath was placed.

**Fig. 2:**
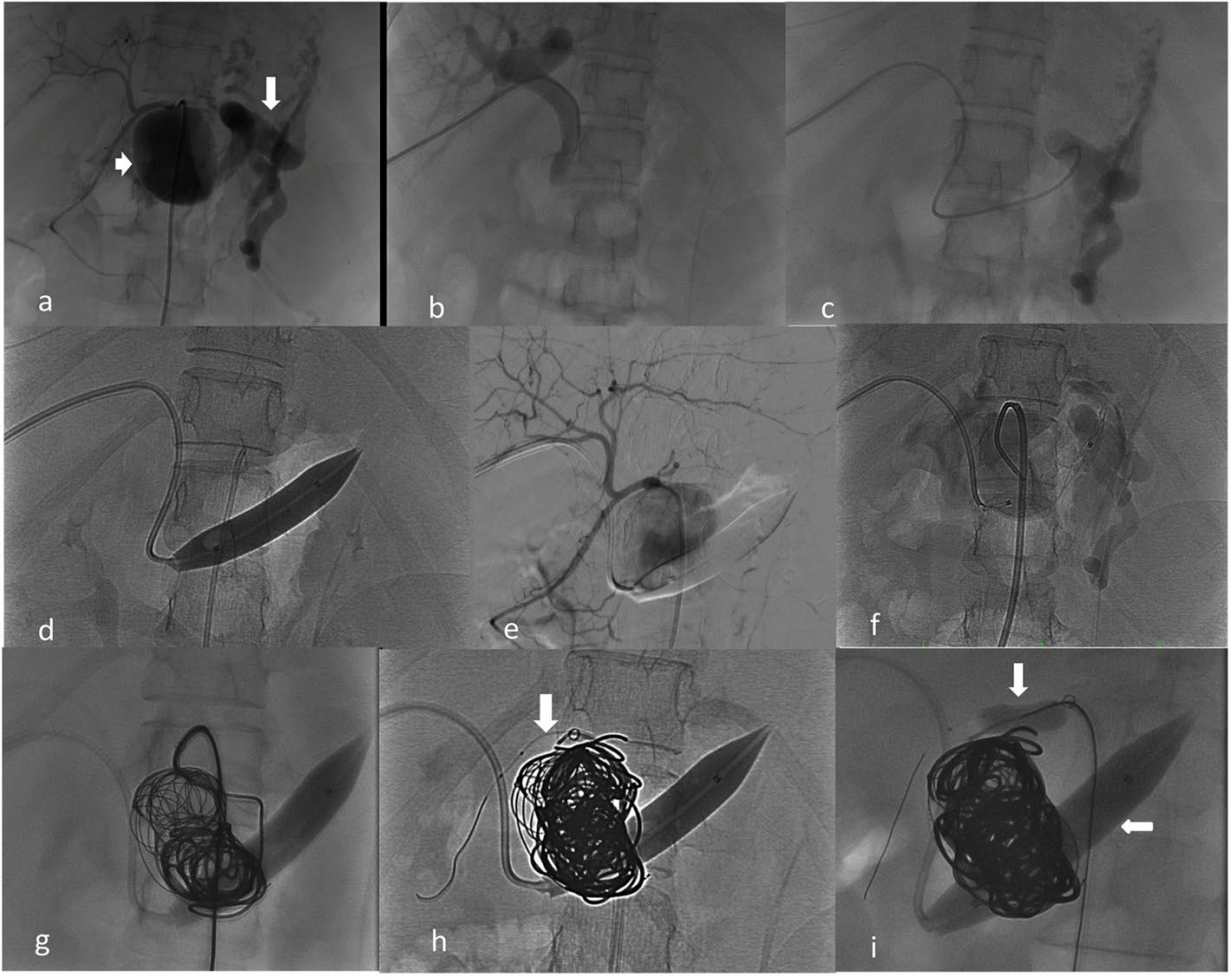
Periprocedural angiograms.** A** Coeliac angiogram demonstrates a splenic artery aneurysm (short arrow) with early arterial opacification of the splenic veins (long arrow), confirming the presence of an arteriovenous fistula (AVF). **B** Portal venogram obtained via percutaneous puncture. **C** Selective opacification of the splenic veins through the percutaneous portal venous access. **D** Inflated Mustang balloon across the AVF within the splenic vein, providing venous outflow control. **E** Coeliac angiogram shows non-opacification of the splenic veins, confirming effective venous balloon occlusion. **F** Selective cannulation of the splenic artery aneurysm with a Progreat microcatheter. **G** Coil embolisation of the aneurysm sac performed under venous balloon inflation. **H** A second balloon (Eclipse, Balt) (arrow) positioned at the coeliac–hepatic artery bifurcation to achieve arterial inflow arrest. **I** Further coil deployment and glue injection performed under dual-balloon inflation

Dual-balloon protection was established using a 6 × 15 mm Eclipse (Balt) balloon at the hepatic–coeliac bifurcation (arterial inflow control) and a 12 × 40 mm Mustang (Boston Scientific) balloon in the splenic vein (venous outflow control). Only after dual-balloon inflation, contrast opacification of the complete SAA and the splenic artery was seen. Through a Progreat microcatheter, the aneurysm sac was densely packed with eight detachable coils (Interlock 2D, Nester, Spirales) with proximal landing of the coils in the splenic arterial stump, followed by injection of a 50% NBCA-Lipiodol mixture under dual-balloon occlusion with percolation also achieved in the splenic artery for a short distance distal to the aneurysm (Fig. 2).

Post-embolisation angiography after balloon deflation confirmed complete exclusion of the aneurysm and fistula with preserved portal venous flow and hepatic arterial flow. Portal venous puncture tract was embolised using pushable coils and glue.

Patient remained haemodynamically stable throughout the procedure, with no non-target embolisation or access site complications. She was discharged after 48 h.

**Fig. 3:**
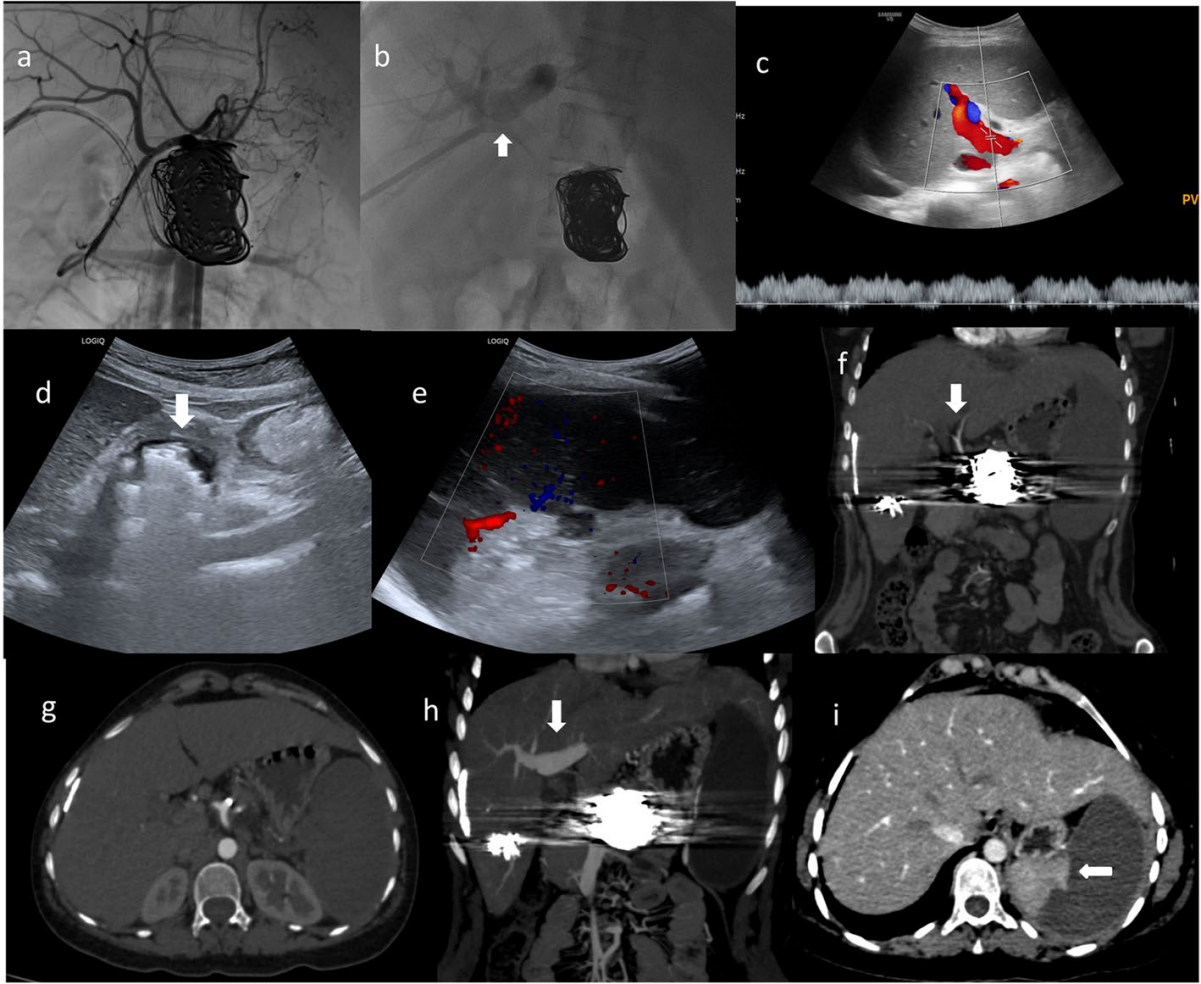
Post-embolisation imaging. Post-embolisation angiograms (**A**, **B**). **A** Coeliac angiogram shows complete exclusion of the arteriovenous fistula with normal opacification of the common hepatic artery. **B** Normal opacification of the portal vein (arrow) and its branches with the coil mass in situ. Follow-up ultrasound of the abdomen (**C**–**E**): **C** Portal venous Doppler demonstrates normal hepatopetal flow with preserved phasic variation. **D** Hyperechoic focus in the epigastrium corresponding to the coil mass (arrow). **E** Colour Doppler shows preserved arterial flow in the upper pole of the spleen. Follow-up CECT (**F**–**I**): **F**, **G** Arterial-phase images demonstrate normal enhancement of the hepatic arteries with absence of early contrast opacification of the portal and splenic veins, indicating complete closure of the arteriovenous fistula. **H** Portal-venous-phase image shows normal contrast opacification of the portal vein (arrow). **I** Enhancing splenic parenchyma in the superior pole (arrow), consistent with viable splenic tissue

Seven-day follow-up Doppler ultrasonography revealed patent portal flow and a thrombosed aneurysm sac. At 15 day follow-up CECT, normal contrast opacification of the common hepatic arteries, portal and hepatic veins was seen with no evidence of early arterial-phase enhancement of the portal and splenic vein, confirming successful closure of the arteriovenous fistula. Arterial enhancement was seen in the superior pole of spleen, consistent with preserved viable parenchyma. The patient remained asymptomatic and clinically stable (Fig. 3).

## Discussion

SAVFs may present incidentally or with manifestations of portal hypertension [3, 4] and cardiac overload. Timely recognition and intervention prevent rupture and haemodynamic complications. EV treatment has been shown to provide comparable technical success to surgery but with lower mortality (0% vs > 4%) [2], and faster recovery.

The 2023 *Radiology Case Reports* study by Alexander and Santos [5] described the first SAVF treated using both coils and NBCA, achieving durable occlusion but with splenic infarct as a complication. The combination of coils (mechanical scaffolding) and NBCA (permanent rapid occlusion) enhances durability but demands meticulous flow control in high-flow shunts to prevent non-target embolisation and to preserve portal haemodynamics. Das et al. [6] encountered coil migration highlighting inadequate flow control. They adopted a strategy with arterial balloon occlusion to circumvent it.

Balloon-assisted techniques for wide-neck SAAs permit controlled, dense coil packing while protecting the parent vessel and reducing coil migration, splenic infarction, and post-embolisation syndrome [7].

Our case applied these principles to a high-flow SAA–SAVF by using *dual-balloon flow arrest*—arterial inflow control at the hepatic–coeliac bifurcation and venous outflow control within the splenic vein—to create a quiescent field for dense coil scaffolding followed by NBCA deposition. This extends the *neck-remodeling* principle to both arterial and venous compartments, minimising non-target embolisation, enabling precise glue polymerisation while preserving portal venous and hepatic arterial flows.

This dual-balloon-assisted approach conceptually parallels the *‘sandwich embolisation’ technique* described by Piechowiak et al. (AJNR, 2017) [8] for dural AVFs of the transverse–sigmoid sinuses, wherein transarterial balloon-assisted Onyx injection was performed under simultaneous transvenous sinus balloon protection, creating a controlled flow-arrest chamber that enabled targeted embolic penetration and minimised reflux into normal venous channels. By adapting this neuroendovascular paradigm to a visceral high-flow shunt, our dual-balloon approach allowed safe, dense coil packing and precise NBCA delivery. To the best of our knowledge, this is the first reported case in the literature of coil and NBCA embolisation of an SAA–SAVF performed under dual-balloon flow control.

An additional important consideration in this case was the patient’s several clinical features—short stature, cachexia, micrognathia, and a large visceral aneurysm at a young age—suggested an underlying connective tissue disorder, most likely vascular Ehlers–Danlos syndrome (vEDS) or a related vasculopathy. Patients with vEDS have inherent arterial fragility due to *COL3A1* gene mutations, with high rates of spontaneous dissections, aneurysms, and ruptures [9, 10]. Awareness of this possibility was critical for procedural planning [9]. In such individuals, aggressive catheter manipulation, high-pressure injections, or extensive hardware exchanges can precipitate vascular rupture [11]. Therefore, we employed a gentle, low-pressure, minimal-instrumentation strategy with balloon-assisted flow control to ensure safety while achieving complete embolic exclusion. Although genetic confirmation of vEDS was not feasible, definitive diagnosis requires COL3A1 mutation analysis. Systemic vascular surveillance, including thoracic aortic imaging, is recommended in suspected vEDS and has been advised as part of longitudinal follow-up, as thoracic imaging was not clinically indicated during the index admission.

This case thus underscores not only the technical innovation of dual-balloon-assisted coil and NBCA embolisation but also its adaptability for patients with suspected connective-tissue disorders, where procedural safety margins are significantly narrower.

## Conclusion

Dual-balloon-assisted coil and NBCA embolisation provides precise flow control and safe, effective treatment of high-flow SAA–SAVF, particularly valuable in patients with suspected connective-tissue disorders such as vEDS. This strategy enhances procedural safety, reduces non-target embolisation, and preserves hepatic arterial, splenic, and portal venous physiology.

## Data Availability

The data and material will be available upon request.

## Abbreviations

SAA: Splenic artery aneurysm
SAVF: Splenic arteriovenous fistula
NBCA: N butyl cyanoacrylate
vEDS: Vascular ehler danlos syndrome
